# Bone-Healing Pattern on the Surface of Titanium Implants at Cortical and Marrow Compartments in Two Topographic Sites: an Experimental Study in Rabbits

**DOI:** 10.3390/ma12010085

**Published:** 2018-12-27

**Authors:** David Soto-Peñaloza, Marco Caneva, José Viña-Almunia, José Javier Martín-de-Llano, David Peñarrocha-Oltra, Miguel Peñarrocha-Diago

**Affiliations:** 1 Oral Surgery and Implant Dentistry Division, Stomatology Department, Faculty of Medicine and Dentistry, University of Valencia, 46010 Valencia, Spain; dr.davidsotpe@gmail.com (D.S.-P.); josevinaalmunia@gmail.com (J.V.-A.); miguel.penarrocha@uv.es (M.P.-D.); 2 ARDEC Academy, Ariminum Odontologica, 47932 Rimini, Italy; marco.caneva@tiscali.it; 3 Department of Pathology and Health Research Institute of the Hospital Clínico (INCLIVA), Faculty of Medicine and Dentistry, University of Valencia, 46010 Valencia, Spain; j.javier.martin@uv.es

**Keywords:** animal study, bicortical stabilization, implant macro-design, osseointegration, dental implant

## Abstract

This study evaluates the bone-healing patterns on the surface of titanium implants at the cortical and marrow compartments of bicortically-installed implants in the diaphysis and metaphysis of rabbit tibiae. In 27 New Zealand rabbits, two implants, one for each macro-design and with equal resorbable blasted media (RBM) implant surfaces, were randomly implanted in the diaphysis or metaphysis of each tibia. The flaps were sutured to allow submerged healing. The animals were sacrificed after two, four, or eight weeks, with nine weeks used for the period of healing. Ground sections were prepared and analyzed. No statistically significant differences were found between the two groups for newly formed bone in contact with the implant surface after two, four, and eight weeks of healing. Bone apposition in the marrow compartment was slightly higher in the diaphysis compared to metaphysis regions across healing stages. Despite the limitations of the present study, it can be concluded that new bone apposition was better than average in the cortical compartment as compared to the marrow compartments. Bone morphometry and density may affect bone apposition onto the implant surface. The apposition rates were slightly better at both the cortical and marrow compartments in diaphysis as compared to metaphysis sites. The new bone formation at the marrow compartment showed slightly better increasing values at diaphysis compared to metaphysis implantation sites.

## 1. Introduction

Osseointegration is a dynamic process strongly influenced by the implant surface, which plays a role during the early phase of healing through resorptive and appositional events [1,2,3]. Among factors that may exert an effect on bone-to-implant interfacial remodeling and new bone apposition [4], the host bed characteristics have been reported as a relevant factor [3,5,6]. 

For this process to be successful, good primary stability is required [7]. The mechanical interlocking that occurs between the surface of the implant and the parent bone in part depends on the implant macro-design, the roughness of the surface, and the surgical preparation of the implant bed [8]. Also, recent evidence reinforces the concept that the implant geometry and the density of the bone are key factors involved in the degree of primary stability [9].

Dental implant surface modifications provide different topographical characteristics and increase the tridimensional surface area [10]. The area is quantified through the surface roughness features, which may be reported as R_a_ or S_a_, depending on whether the parameter is classified as two-dimensional or three-dimensional [11]. Modified-surface implants provided enhanced activation of the platelets and the cell adhesion and protein adsorption relating to the implant surface [10,12]. These features improve the osseointegration process in terms of bone-to-implant contact (BIC) and implant stability during the early healing phase [13], and may up-regulate osteogenic activity and osteoconductivity [12,14].

The improvement of the bioactivity of dental implants, so they could be able to respond to body fluids, cells, and pathogenic agents, was a result of the different approaches used to produce multifunctional Ti-based surfaces [15]. This is a result of the implementation of inorganic coatings, chemical surface modifications, and bioactivation by means of organic coatings [15]. Several methods for implant surface treatments (e.g., etched surfaces, sandblasted and acid etched surfaces, hydroxyapatite-coated surfaces, grit-blasted surfaces, laser ablation, fluoride treatment) have been introduced throughout the years [16]. Among them, the resorbable blasted media (RBM) surface is obtained through the grit-blasting of calcium phosphate bioceramic (CaP) particles at high velocity, in which the particle size determines the roughness degree as a particle-free titanium surface [17,18].

A more homogenous honeycomb-like spatial distribution with a lower roughness has been reported for RBM surfaces [19,20]. Also, the RBM surfaces showed BIC values that were comparable to other blasting surfaces, such as titanium dioxide (TiO_2_) or aluminum oxide (Al_2_O_3_) [21], and similar biomechanical strength and removal torque measurements were obtained by calcium- and magnesium- enhanced implants [22].

In osseointegration, healing pattern differences between the cortical and the marrow compartments at both flat (dog jaw) and long bones (sheep tibia) were noticed in previous studies [23,24]. This behavior is observed in rabbit tibiae as well, and despite inherent experimental model differences, it differs from former studies because distinct surface treatments were assessed [25]. Indeed, in the study of Caneva et al. 2015 [25], the effects of equal implant geometries have been tested, but at different implant surface modifications.

Therefore, it is presumable that both the cortical and marrow compartments provide distinct biological and physical features at bone-to-implant interfacial remodeling and direct bone apposition toward the implant surface [1,4,26]. Their nature demarcates the transition from primary to secondary/biological stability after an implant stability dip in the osseointegration process [26]. In keeping with these observations, and because of the lack of studies regarding bone-healing pattern on implant surfaces at different bone compartments and bone environments, there is a need for further investigation. Hence, the aim of the present study is to evaluate the bone-healing pattern at the cortical and marrow compartments at equal RBM surfaces of bicortically installed implants in the diaphysis and metaphysis of rabbit tibia.

## 2. Materials and Methods

This preclinical study is performed in abidance with the Animal Research: Reporting In Vivo Experiments (ARRIVE) guidelines [27], and animal selection and use have been carefully considered.

### 2.1. Ethical Statement

The study protocol was approved by the Ethics Committee of Valencia University, Valencia, Spain (Protocol ref.: A1432625410189), which followed the guidelines established by the Council Directive of the European Union (53/2013; February 1, 2013) for animal care and experimentation in agreement with the ethical and legal conditions established by Royal Decree 223, March 14 and October 13, 1988.

### 2.2. Study Design and Experimental Animals

The experimental pre-clinical study involved twenty-seven males, albino New Zealand rabbits, 24 weeks of mean age and weighing 3–4 kg. The animals were segmented into three groups composed of 9 animals each and sacrificed at 2, 4 and 8 weeks, respectively. Implants were put into the animals in a random allocation, a resulting in the imposition of four dental implants in each rabbit; two in each tibia, one in the diaphysis, and the other in the metaphysis.

### 2.3. Randomization and Allocation Concealment

Before surgery, the animals were put in one of the three groups by random allocation, each group representing a healing period. Two implants each with a different macro-design were installed in each tibia. The position of each implant, i.e., the diaphysis or metaphysis, was randomly assigned. The aleatory choice was carried out electronically (www.randomization.com) by an independent author neither involved in the selection of the animals nor in the surgical procedures.

### 2.4. Implant Features

Ticare^®^ implants (Mozo-Grau, Valladolid, Spain) made of commercially available pure grade-IV titanium treated with resorbable blast media (RBM) (implant surface blasted with calcium phosphate ceramics, resulting in a moderately rough (R_a_ = 1.53 ± 0.24) surface) were used. All implants had a dimension of 3.75 mm of diameter and 8 mm of length and a conical connection with a 45° polish neck with a self-tapping feature closer to the apex.

### 2.5. Surgical Procedures

**Figure 1:**
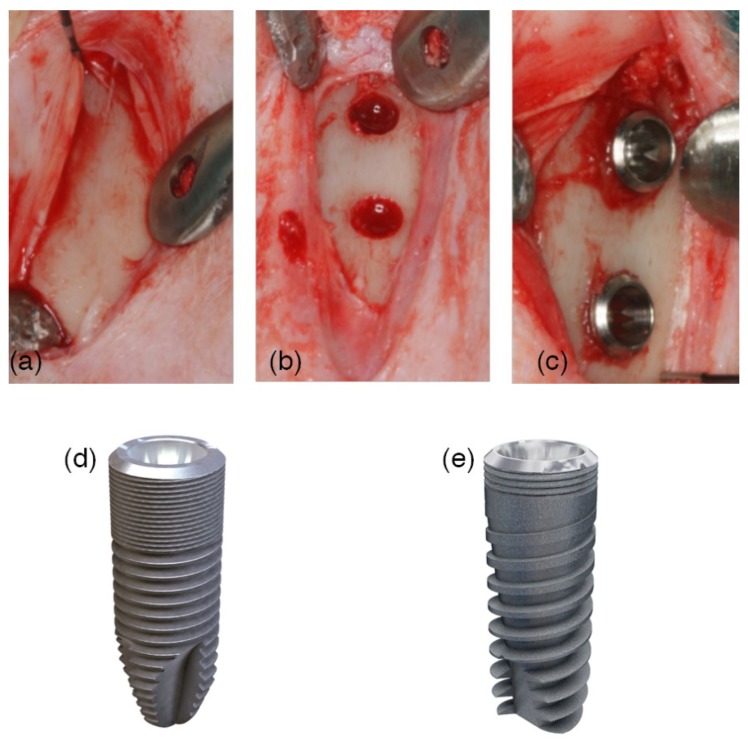
A surgical image. (**a**) An incision in the skin was performed at the medial section of the tibia. The surgical flaps were raised, showing off the proximal area of the tibia. (**b**) Two implantation sites were prepared with the same drill protocol, at the metaphysis and the diaphysis areas. (**c**) Randomly, two implants with different macro-designs were installed and separated by a distance of 8 mm in between. (**d**) Ticare Inhex**^®^:** The implant body had a little conicity and a large area of micro-threads at the coronal portion, and a higher number of triangular threads per unit length and with little thread depth compared to the Quattro^®^ model. Moreover, the implant featured a double self-tapping at the apical portion. (**e**) Ticare Inhex Quattro^®^: The implant body had a marked conicity. Fewer micro-threads at the coronal portion and a lower number of macro-threads were present as compared to Ticare Inhex^®^ implants. The threads were squared in the middle part of the implant and became triangular and deeper at the apex, with aggressive self-tapping at the apex.

The rabbits were induced to anesthesia with Ketamine injection (22 mg/kg) intramuscularly, xylazine (2.5 mg/kg) and intravenous injection of Propofol (1.5 mg/kg) and maintained with 2% of isoflurane. Before surgery, the rabbits’ fur that was proximal to the tibia was shaved and disinfected with Betadine. A preoperative antibiotic Enrofloxacin 5 mg/kg (ALSIR^®^ 2.5%, Esteve Veterinaria, Barcelona, Spain) was infiltrated subcutaneously, and 3 mL of articaine at 2% with 0.01 mg/mL epinephrine infiltrative anesthesia was intramuscularly applied in the surgical area of each leg. The skin of both tibiae was incised in the proximal region, the flaps were raised, and the bone was shown below the anterior tibial tuberosity (Figure 1a). Both areas, one in the metaphysis and the other in the diaphysis, were identified as experimental sites. The recipient sites were prepared following the recommendations of the manufacturer using drills with increasing diameters under irrigation with sterile saline (Figure 1b). A distance of about 8–10 mm was maintained between the two osteotomies (Figure 1c). Two implants with different macro-designs were randomly installed in each tibia: (a) Ticare Inhex^®^ and (b) Ticare Quattro^®^ (Figure 1d,e). The implants were screwed until the implant shoulder was leveled with the bone surface. The implants’ apex was placed in close contact with or into the cortical bone opposing the coronal cortical compartment, facing forward to obtain a bi-cortical anchorage. Cover screws were placed on the implants, and the flaps were subsequently sutured in layers with resorbable sutures (Vicryl 5/0, Ethicon, Sommerville, NJ, USA), and Nylon (Ethilon 3/0, Ethicon, Sommerville, NJ, USA).

### 2.6. Post-Operative Care, Housing and Husbandry

Each animal had its own cage; the room in which they were kept was purposely designed so it could have 12 h of light, and so it was an acclimatized space**.** The animals were fed with a standard diet and had free access to water. The analgesic pattern consisted in 2.5 mg/kg of morphine intraoperative, 0.02 mg/kg buprenodale, buprex, 0.2 mg/kg meloxicam (every 12 h over 3 days) and antibiotic therapy with Enrofloxacin 2.5 mg/Kg (ALSIR^®^ 2.5%, Esteve Veterinaria, Barcelona, Spain) (every 24 h over 7 days) post-operatively. Within 2–3 days, the animals continued to act in a normal way, lacking pain or distress symptoms. Also, after the operation, the wounds were constantly inspected and cleaned with Betadine to prevent future complications.

### 2.7. Euthanasia

The euthanasia of the animals took place at different healing times according to the group; some animals were sacrificed after 2 weeks while others after 4 or 8 weeks. Sacrifice was performed by using the same protocols used for surgery; 50 mg/kg intravenous sodium pentobarbital was applied to each rabbit. Both animals’ tibias were removed, while the adhering soft tissues were dissected. A small electric saw was used to obtain the sections of the tibia containing each implant.

### 2.8. Histological Preparation

Implant samples were dehydrated by sequential solvent exchange and embedded in methyl methacrylate containing poly-(methyl methacrylate). After adding benzoyl peroxide (1 g/100 mL), samples were polymerized at room temperature for several days and were then sawed using a diamond wheel on a precision table top cut-off machine Accutom-5, (Struers, Copenhagen, Denmark) and then were wet ground and polished using a LaboPol-21 system (Struers, Copenhagen, Denmark) and SiC foils. Approximately 80 μm thin sections were obtained using SiC foils of decreasing particle size. The samples were stained at 55 °C with toluidine blue for 30 min, washed with tap water for 2 min, and let dry.

### 2.9. Histological Examination

Overlapping calibrated digital images of the tissues surrounding the whole implant surface (about 20 images/implant) were recorded with a bright field Leica DM4000 B microscope (Leica Microsystems GmbH, Wëtzlar, Germany) and DFC420 digital camera using a 5× objective and the Leica Applications Suite version 4.4.0 software. Individual images were merged to compose each implant side using the Photoshop program (Adobe Photoshop CC 2015.0.0, Adobe Systems Incorporated, San José, CA, USA; http://www.adobe.com/Photoshop). The image processing program ImageJ 1.48 (National Institutes of Health, Bethesda, MD, USA; http://imagej.nih.gov/ij) was used for histological measurements. Lines were drawn by hand on calibrated images showed on the computer screen at 400× magnification by an independent and calibrated assessor not involved in the study.

**Figure 2:**
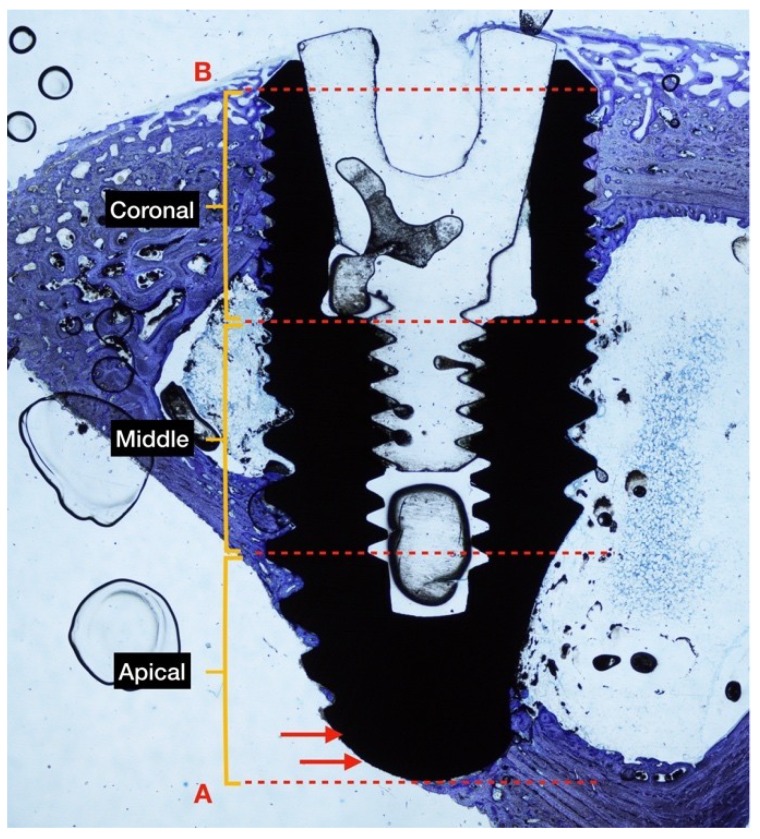
The ground section of rabbit tibia in diaphysis position at 4 weeks of healing. The implant is divided into 3 equal sections (coronal, middle, apical) for BIC measurement. Two points were traced: (B) The most coronal part of the bone to implant contact, and (A) the base of the implant. The implant surface outside of the cortical bone is not considered in the analysis (red arrows). Original magnification ×2 was used, as was toluidine blue staining.

The following reference highlights were traced to identify: (B) The most coronal bone-to-implant contact and (A) the base of the implant. Three sections with similar lengths were established to divide the implant within coronal, middle, and apical areas regarding the long axis [25]. The percentages of (nb) new bone, (ob) old bone, and (m) bone marrow in contact with the implant surface were measured on the entire implant length as well as on each of the three sections. The BIC was examined as the sum of new and old bone, and percentages in relation to the length of the implant surface were examined calculated. The apical portion of the implant that extruded beyond the compact cortical layer was excluded from the analyses (Figure 2).

### 2.10. Data Analysis

Differences between bone compartments throughout the follow-up post-operation process were analyzed with the Mann–Whitney U-test for independent variables. Differences between implants placed in the diaphysis and metaphysis, respectively, were also performed using a Wilcoxon rank-sum test. The level of significance was set at a = 0.05. The data employed in the present work were used in another study but were focused at the implant macro-design level.

## 3. Results

### 3.1. Clinical and Histological Outcomes

All surgeries were performed in an operating room within the Central Unit of Investigation in Medicine (UCIM) at the University of Valencia. No complications occurred during the healing period. All implants seemed adequately integrated into the histological evaluation across each period. Finally, the data of 27 experimental animals with four implants each were analyzed.

The areas between the threads were filled with woven bone at two weeks. Remodeling processes were observed after 4 and 8 weeks of healing, as shown by the lighter-staining of the lamellar bone compared to the darker-staining of the woven bone. The bone healing stages follow the intramembranous-type and appositional ossification mode patterns. The latter could be observed where intimate contact between the implant surface and newly formed bone from the implant bed occurred. There were no significant differences regarding new osseointegration values and mineralized bone to implant contact (new bone + old bone) for both implant macro-designs assessed and between implants placed at diaphysis or metaphysis implantation sites (*p* > 0.05) (data not shown).

On average, better osseointegration values were identified in the cortical compartments, and slightly higher but no statistically significant values at the diaphysis sites. Regarding the marrow compartment, better apposition rates of new bone were observed at two and four weeks at the diaphysis sites. The parameters assessed regarding its position (diaphysis or metaphysis) and compartment (cortical or marrow) are depicted in Table 1.

### 3.2. 2-Weeks of Healing

**Figure 3:**
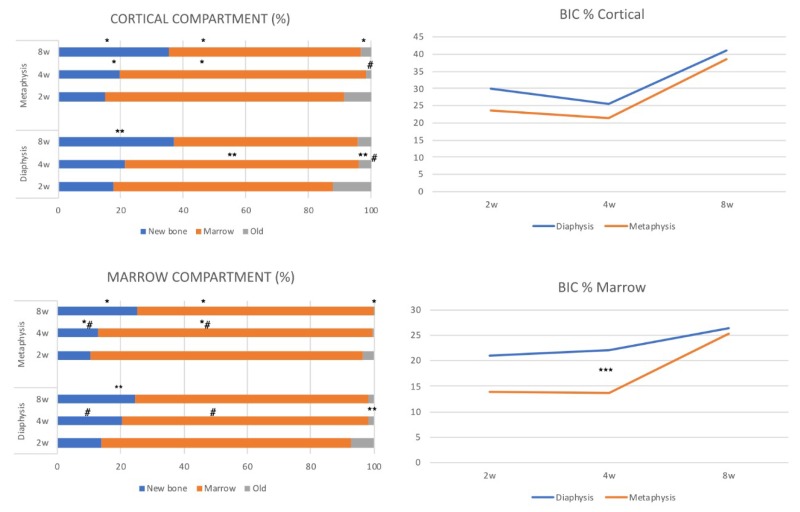
The total amount of new bone, marrow content, and old bone according to cortical (**a**) and marrow (**b**) compartment for implants placed via either diaphysis (n = 9) or metaphysis (n = 9) at different healing periods. Mineralized bone to implant contact (new + old) for cortical (**b**) and marrow (**d**) compartments at the various healing times. *p* < 0.05; between cortical and marrow compartments at metaphysis sites (*), at diaphysis sites (**) or (#) between diaphysis and metaphysis within cortical and marrow compartments and (***) for mineralized bone to implant contact, respectively. Comparisons are based on the same healing stage in weeks (w).

**Figure 4:**
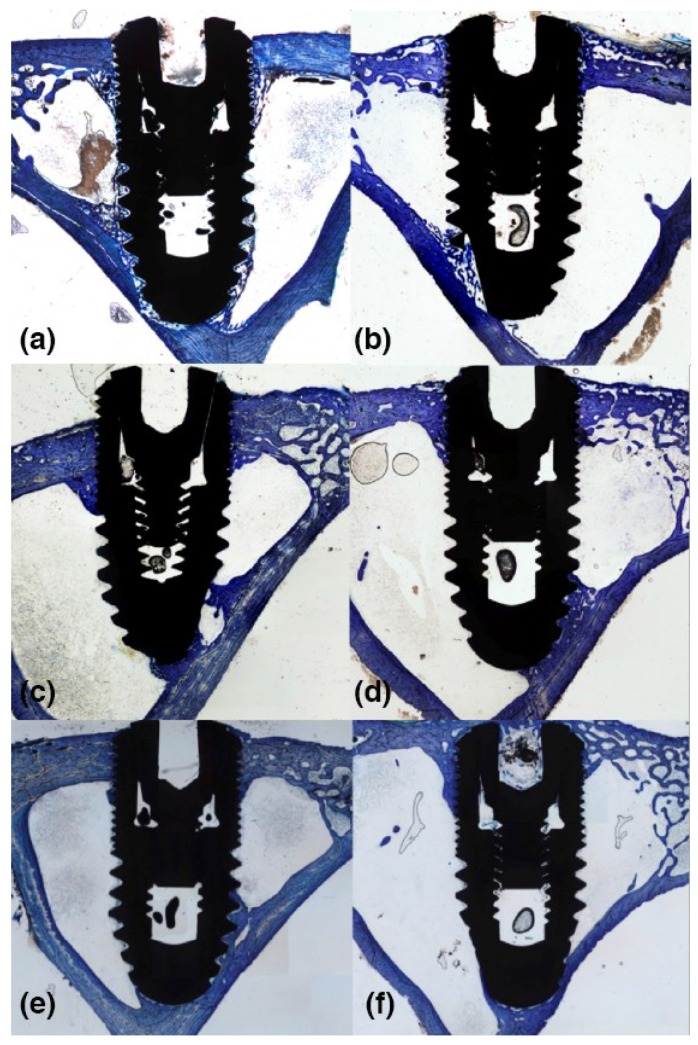
Ground section of bicortically-installed implants (Ticare Inhex^®^, Mozo Grau, Valladolid, Spain) at diaphysis (left side) and metaphysis (right side) sites at two (**a**,**b**), four (**c**,**d**) and eight (**e**,**f**) weeks of healing. An original magnification of ×2.5 and toluidine blue staining was used.

There were not significant differences among the parameters assessed between the cortical and marrow compartments in both the diaphysis and metaphysis sites (Table 1; Figure 3a,c). BIC% values (Figure 3b,d) were around 30 ± 9.9% versus 23.7 ± 6.4% for diaphysis and metaphysis sites respectively in the cortical compartment (*p* = 0.09), and 21.1 ± 12.3 versus 13.9 ± 8.0 in marrow compartment (*p* = 0.07). Ground sections for the diaphysis and metaphysis at this stage are shown in Figure 4a,b.

### 3.3. 4-Weeks of Healing

Significant differences were observed at this stage of healing for old bone at cortical compartment and for new bone and soft tissue in the marrow compartments between diaphysis and metaphysis sites (Table 1, Figure 3a,c). No differences were detected for BIC% values in the cortical compartment (Figure 3b) that were around 25.4 ± 7.8% and 21.4 ± 8.0% for diaphysis and metaphysis sites, respectively (*p* = 0.26). However, a significant difference was found in the marrow compartment (Table 1, Figure 3a,c), showing BIC% values of 22.1 ± 6.9 and 13.6 ± 8.5 (*p* = 0.01) for diaphysis and metaphysis sites, respectively (Figure 3d). Ground sections for the diaphysis and metaphysis at this stage are presented in Figure 4c,d.

### 3.4. 8-Weeks of Healing

On average, better values for new and old bone were observed in the cortical compartment; a significant difference was detected between the cortical and marrow compartments for these parameters in metaphysis sites (Table 1, Figure 3a,c). The mineralized bone-to-implant contact at this stage did not show significant differences within the cortical compartment between diaphysis and metaphysis implant sites, with BIC% values 41.1 ± 6.8% and 39.9 ± 9.8%, respectively (*p* = 0.61). A similar trend was observed within the marrow compartments at diaphysis and metaphysis sites (Figure 3b,d). Ground sections for diaphysis and metaphysis at this stage are presented in Figure 4e,f.

## 4. Discussion

The present study evaluates the bone-healing patterns at cortical and marrow compartments at equal surface bicortically-installed implants in the diaphysis and metaphysis of rabbit tibiae. Dental implants were placed in two topographic zones, one with a cortical layer and a medullar content (diaphysis) like a type II bone, and the another was more trabecular like a type III bone (metaphysis).

The histomorphometric analyses at two, four and eight weeks showed no differences (*p* > 0.05) for either of the implant macro-designs. The new bone and BIC percentages in relation to the topographic implant placement after four and eight weeks of healing showed that osseointegration tends to be slightly higher but is statistically less significant for implants placed in the diaphysis than the metaphysis sites, with BIC values of 24.5 ± 6.2% and 18.4 ± 7.7% at four weeks; and 41.1 ± 6.8% and 39.9 ± 9.8% at eight weeks. After a month of healing, old bone still remains (<3%). This result is similar to some previous preclinical studies which have already highlighted this finding [1,24,25], and in humans [28,29].

As reported by previous studies, it is known that resorptive processes occur before new bone apposition in zones where the mineralized bone is present, conveying a slightly longer healing period to reach complete osseointegration [1,30]. Indeed, in the present study, the new bone formation in the marrow compartment showed slightly better increasing values of 13.8%, 20.4, and 24.6% at two, four and eight weeks at diaphysis sites, compared to 10.3%, 13%, and 25.1% at metaphysis sites. The same trend was observed in the cortical compartment with values around 17.8%, 21.4%, and 37% at diaphysis sites, and 15.1%, 19.7%, and 35.5% in metaphysis sites. The old bone was resorbed but was still present (<2%) after 1 month in both topographical zones. The parent old bone values observed were slightly higher in diaphysis implant sites at four and eight weeks compared to metaphysis sites in the present study sample.

However, in this study, it is possible to appreciate a contradiction of what was established by the study of Caneva et al. 2015 [25]. In this study, the new bone formation developed at a much higher speed at the implants placed in the metaphysis that those in the diaphysis. The authors attributed the findings to the denser pattern of the trabecular bone in the metaphysis compared to the diaphysis tibiae, an event that may have empowered osseointegration. On the other hand, the bone formation that was supposed to be reinforced by the bone marrow did not work out, since this scarcely contributed to its formation in the middle section of the implants placed in the diaphysis compared to what was found in the coronal and apical sections.

These observations could be attributable to several factors in study design, such as the implant thread designs, the different surface treatments tested, and the implant osteotomy protocols differing between studies. These are factors that could regulate the strain applied to hard tissue in proximity to the implant [31,32]. Also, in the study of Caneva et al. there were demarcated three sections (coronal, middle, and apical) to test the differences among compartments [25]. In the present study, the three sections were demarcated in the same manner, but the cortical compartment is considered as the sum of the cortical and apical regions as a whole, independently of the marrow compartment (middle). In both studies, the extreme regions of the implants were in close contact with pristine bone (bicortical stabilization), a condition that may have reinforced osseointegration on the implant surface [25].

The parent old bone in the recipient site is responsible for mechanical interlocking, and thereafter for cell-mediated interfacial bone remodeling [4,26]. This is known to typically occur in the area of contact between the bone wall of the implant bed preparation and the implant surface, where remodeling arises in the proximity of microcracks, subsequently followed by new bone apposition in void spaces resulting in secondary or biological stability [4].

Notably, the bone morphology in diaphysis sites is predominantly occupied by a marrow content in comparison to the metaphysis, which presents more trabecular bone. These findings are in agreement with the premise that osseointegration is faster in areas where bone apposition is not precluded by bone resorption, based on results in the dog model [1] and further confirmed in another study in minipigs [33].

Also, it is possible to conclude that osseointegration seems to be favored by the existence of a blood clot, and prejudicated by the presence of the yellow fatty bone marrow in the long bone model, such as sheep tibiae [24]. In this sense, Morelli et al. 2014 employed the sheep tibia model, where two osteotomies for implant installation are prepared in each tibia. On average, new bone apposition was better in the cortical compartment, as seen in the present study [24]. Moreover, it was observed that new bone apposition was faster in the fatty bone marrow group compared to blood clot groups at marrow compartments after 4 weeks. The authors concluded that osseointegration appeared to be favored by blood clots, because at 12 weeks of healing the test group showed better new bone values, statistically significant only at the marrow compartment [24], even though in this study the implants were not placed bicortically.

However, despite inter-species differences impeding direct comparisons, there is no certain data on the extent to which the rabbit tibia model, in its diaphysis or middle shaft, provides amounts of fatty bone marrow that may affect osseointegration after eight weeks of healing. So, it would be of interest to isolate its effect in a further study in order to confirm this hypothesis in the rabbit tibia model.

The present findings determine, in accordance with previous observations, that there are no differences in BIC rates under the absence of loading conditions [34,35], independently of the implant macro-design. So, the absence of a functional load reflects only the bone-to-implant structural connection, but not the functional properties of the bone to implant interface [34]. It is important to consider that the present results can only be extrapolated to implants with the same surface roughness; their applicability is inappropriate for other animal models because of the differences shown via healing patterns among species (e.g., dogs, sheep, rabbits, and rats). Further studies comparing equal surfaces of treatment but with different manufacturing approaches are warranted to determine if the patterns observed in the present work are replicated.

The novelty of the present work lies in the fact that there is no other study aiming to assess the healing pattern between cortical- and marrow-compartments using two different macro-designs with equal moderately rough surface treatments in two topographic sites. This allows us to isolate the macro-design effects on osseointegration, thus helping us separately evaluate the dynamics of the healing pattern.

An independent analysis regarding topographic implantation sites was performed. Regrettably, it is difficult to determine to which extent each implant macro-design contributes to these findings observed at the diaphysis or metaphysis sites. The scarce sample did not permit a proper statistical comparison. So, further studies are warranted with a greater sample for this aspect, but this is a tough challenge, taking into consideration the ethical and economical aspects that may be involved in consideration of the replacement, refinement, or reduction (3Rs) criteria for the use of animals in research.

## 5. Conclusions

Despite the limitations of the present study, it can be concluded that new bone apposition was better on average in the cortical compartment compared to the marrow compartments. Bone morphometry and density may affect the bone apposition onto the implant surface. The apposition rates were slightly better at both cortical and marrow compartments in diaphysis compared to metaphysis sites. The new bone formation at the marrow compartment showed slightly better increasing values at the diaphysis sites as compared to the metaphysis implantation sites.

## Figures and Tables

**Table 1:** Proportion (%) of tissue components in contact with the implant surface for either the cortical or the marrow bony compartments at the various time periods in weeks (w). n = 9 per each period of healing.

Compartment	Follow-up	Topographic Site	Statistic	New Bone	Soft Tissue	Old Bone
Cortical compartment	2 w	Cort-dia	Mean	17.8	70.0	12.2
			SD	10.6	8.9	6.6
			Median	14.0	68.2	8.3
		Cort-meta	Mean	15.1	76.3	8.5
			SD	5.8	6.4	6.5
			Median	14.8	78.9	6.2
		Differences	*p*	0.39	0.09	0.11
	4 w	Cort-dia	Mean	21.4	74.6	4.0 *^,#^
			SD	6.9	7.8	3.2
			Median	21.6	74.6	3.0
		Cort-meta	Mean	19.7 *	78.6 *	1.7 ^#^
			SD	8.3	8.0	1.5
			Median	17.3	79.3	1.5
		Differences	*p*	0.57	0.26	0.04
	8 w	Cort-dia	Mean	37.0 *	58.9 *	4.1
			SD	5.7	6.8	2.6
			Median	37.3	58.5	4.3
		Cort-meta	Mean	35.5 *	61.3 *	3.2 *
			SD	8.7	9.8	3.4
			Median	33.8	60.9	2.6
		Differences	*p*	0.63	0.62	0.88
Marrow compartment	2 w	Marrow-dia	Mean	13.8	78.9	7.3
			SD	9.2	12.3	8.9
			Median	13.2	82.8	2.9
		Marrow-meta	Mean	10.3	86.1	3.6
			SD	8.2	8.0	5.1
			Median	9.3	89.5	0.5
		Differences	*p*	0.18	0.07	0.23
	4 w	Marrow-dia	Mean	20.4 ^#^	77.9 ^#^	1.7 *
			SD	6.8	6.9	2.3
			Median	19.7	79.4	0.4
		Marrow-meta	Mean	13.0 *^,^^#^	86.4 *^,^^#^	0.6
			SD	8.2	8.5	0.8
			Median	13.3	86.7	0.0
		Differences	*p*	0.02	0.01	0.16
	8 w	Marrow-dia	Mean	24.6 *	73.6	1.8
			SD	12.9	16.3	3.8
			Median	21.6	78.5	0.0
		Marrow-meta	Mean	25.1 *	74.7 *	0.2 *
			SD	9.6	9.7	0.4
			Median	23.2	75.9	0.0
		Differences	*p*	0.878	0.79	0.25

U Mann Whitney-test: *p* < 0.05; SD, standard deviation; * *p* < 0.05 between cortical and marrow compartment either at diaphysis (Cort-dia vs Marrow-dia) and metaphysis (Cort-meta vs Marrow-meta) topographic regions (vertical); ^#^ *p* < 0.05 between diaphysis and metaphysis either at cortical (Cort-dia vs Cort-meta) or marrow compartment (Marrow-dia vs Marrow-meta) (horizontal).

## Supplementary Materials

Supplementary File 1

## Abbreviations

BIC	bone to implant contact
RBM	resorbable blasted media
CaP	calcium phosphate bioceramic
NB	new bone
ST	soft tissue
OB	old bone
